# Supplementary material for: Analyzing a networked social algorithm for collective selection of representative committees
Source: PLoS One. 2019 Sep 26;14(9):e0222945. doi: 10.1371/journal.pone.0222945 (PMC6763197; doi:10.1371/journal.pone.0222945)
Supplement: S1 Text — (PDF) [file pone.0222945.s001.pdf]

Supplementary Material: Analyzing a networked social algorithm for collective selection of representative committees

Alexis R. Hernández[1*], Carlos Gracia-Lázaro[2], Edgardo Brigatti[1]
and Yamir Moreno[2,3,4]

1 Instituto de Física, Universidade Federal do Rio de Janeiro, Rio de Janeiro, Brazil
2 Institute for Biocomputation and Physics of Complex Systems (BIFI), Universidad de Zaragoza, Zaragoza, Spain
3 Department of Theoretical Physics, Faculty of Sciences, Universidad de Zaragoza, Zaragoza, Spain
4 ISI Foundation, Turin, Italy

* elchechi@gmail.com

1 Modular Networks

In order to study the effect of the modularity of the network, we consider stochastic block model networks [1] with a fixed number of communities n_c. First, we establish a partition of the nodes set into disjoint subsets of communities, so there are not overlapping communities. Subsequently, the network is generated by a generalization of the Erdös-Rényi process where links inside the community have a higher probability to occur: two nodes belonging to the same community are connected with probability p, while two nodes belonging to different communities are connected with probability q. This method allows us to make-up networks for any modularity m, defined as the fraction of the intra-community links minus the expected fraction if links were distributed at random ($p = q$). All the communities have the same number of individuals N/n_c. In Fig. 1 we plot the results for a network with eight communities and modularity $m = 0.4, 0.6, 0.8$. As can be appreciated, the effects of the modularity appear to be marginal both on the representativity and integrity. In Fig. 2 we also

consider different numbers of communities n_c. Even for a high number of communities the effect of the modularity appears to be irrelevant.

Variating the modularity (8 communities)

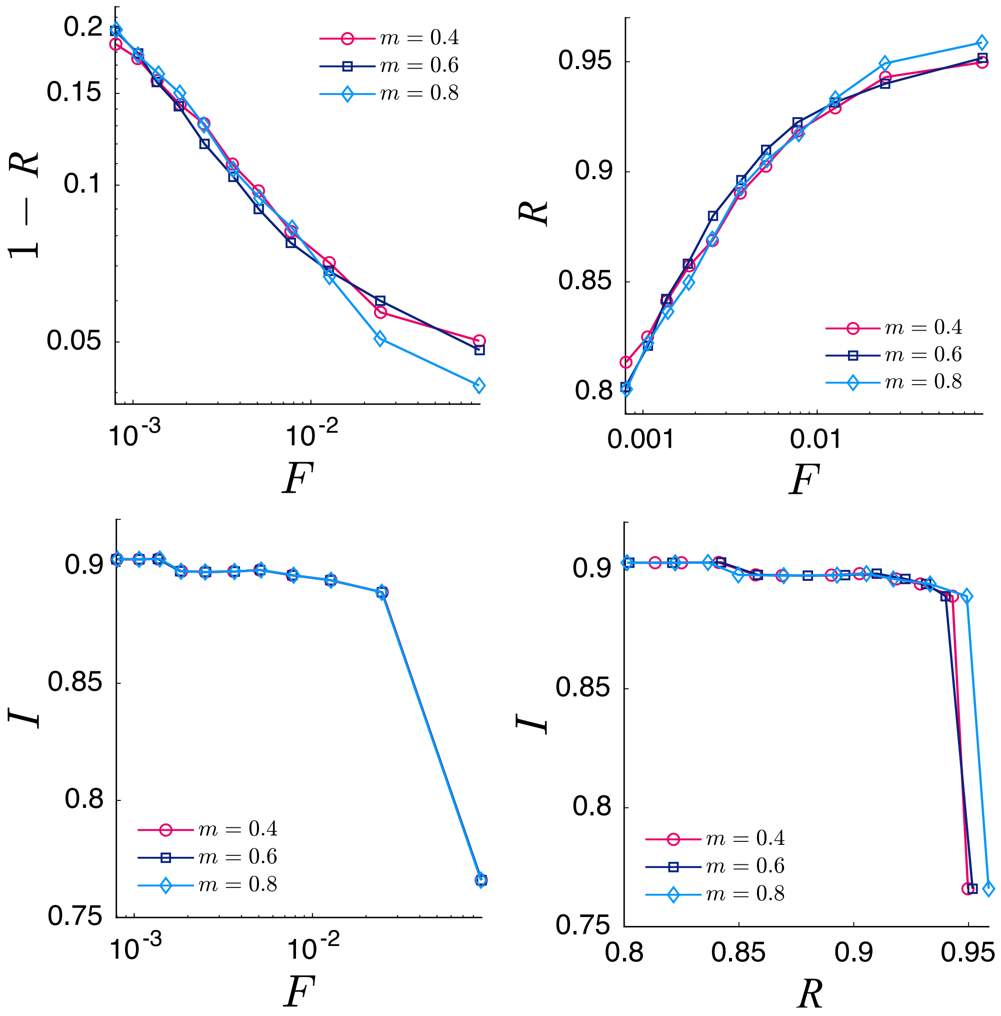

Figure 1. *Top:* On the left, logarithmic plot of $1 - R$ versus normalized committee size. On the right, R versus normalized committee size. *Bottom:* Committee Integrity as a function of normalized committee size (left) and representativity R (right). We consider a network with $N_e = 10000$, 8 communities and modularity $m = 0.4, 0.6, 0.8$, $\langle k \rangle = 40$ and $\sigma^2 = \sigma_p^2 = 0.05$. Results are averaged over 100 different realizations.

Results for networks with different number of communities n_c

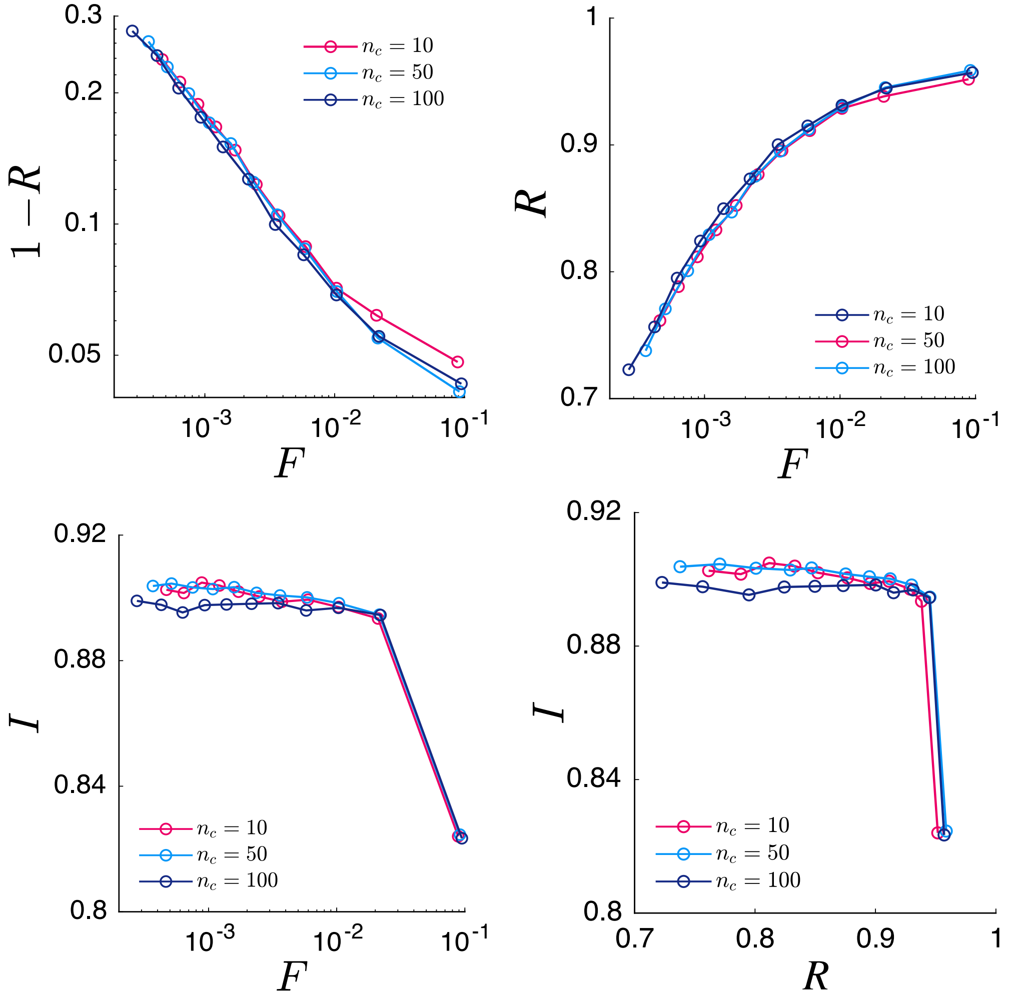

Figure 2. *Top:* On the left, logarithmic plot of $1 - R$ versus normalized committee size. On the right, R versus normalized committee size. *Bottom:* Committee Integrity as a function of normalized committee size (left) and representativity R (right). We consider a network with $N_e = 10000$, $n_c = 10$, 50, 100 communities and modularity $m = 0.8$, $\langle k \rangle = 40$ and $\sigma^2 = \sigma_p^2 = 0.05$. Results are averaged over 100 different realizations.

2 Refusal to participate

In this section we consider a fraction u of individuals which have not disposition to contribute as representatives. In practice, we remove individuals from the committee with a probability u. The results are present in figure 3. Here we appreciate that even for very high fractions of unavailable individuals u the system still behave properly, presenting a small drop on both the representativity and the integrity. The integrity also anticipates the big drop which happens when individuals with a very small amount of votes start entering to the committee. However, we still have a region where good values

of representativity and integrity can be obtained with a reasonable committee size.

Considering a fraction (u) of unavailable individuals

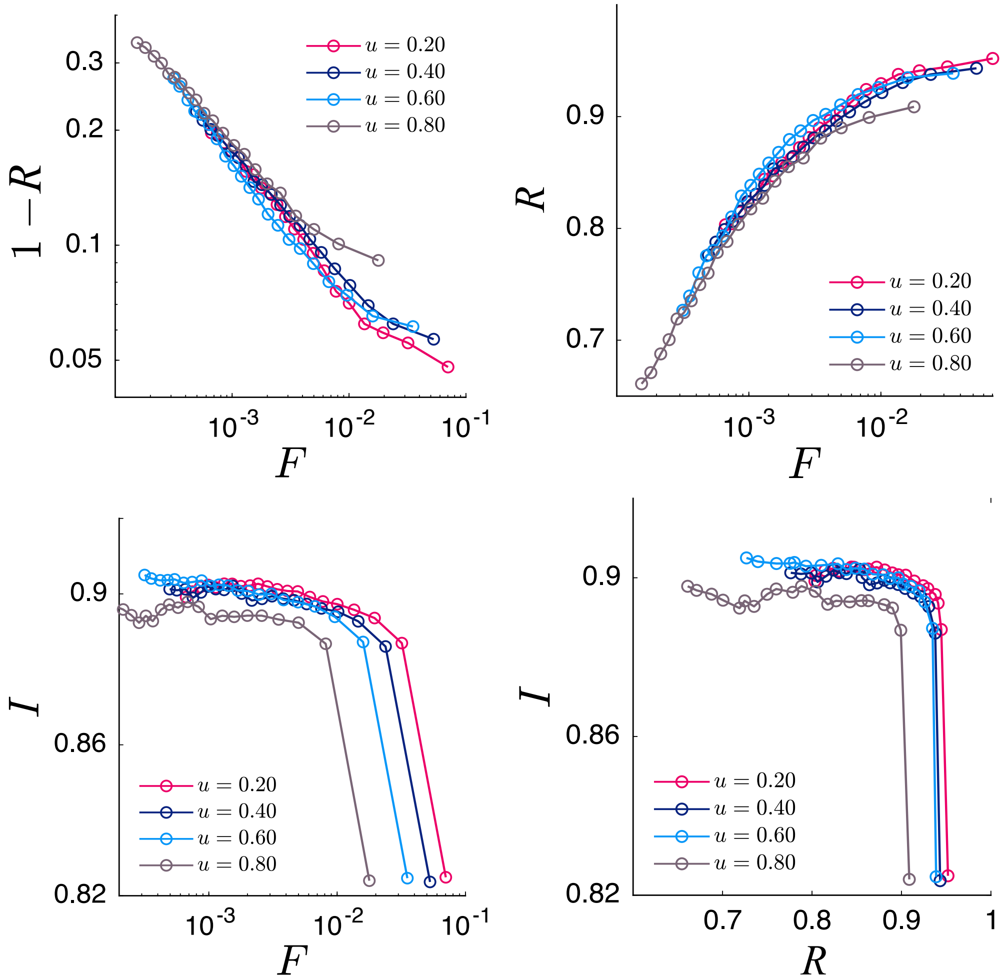

Figure 3. *Top:* On the left, logarithmic plot of $1 - R$ versus normalized committee size. On the right, R versus normalized committee size. *Bottom:* Commitee Integrity as a function of normalized committee size (left) and representativity R (right). We consider a fraction of the individuals (u) as unavailable, a Erdös-Rényi network with $N_e = 10000$, $\langle k \rangle = 40$ and $\sigma^2 = \sigma_p^2 = 0.05$. Results are averaged over 100 different realizations.

3 Self-declared individuals

In this section we consider a fraction of individuals which refuse to transfer the received votes. In practice, it is implemented by modifying the algorithm to allow self indication. Again, the effect on the representativity and the integrity of the resulting committee are very small (Fig. 4). What happens here is that self-declared individuals cut branches on the trees present in the transfer voting network. It produces a different set of

representatives with a smaller amount of votes but still, the probability of entering this group is strongly dependent on the individuals' integrity and the overlap of opinions.

Considering a fraction (s) of self declared candidates

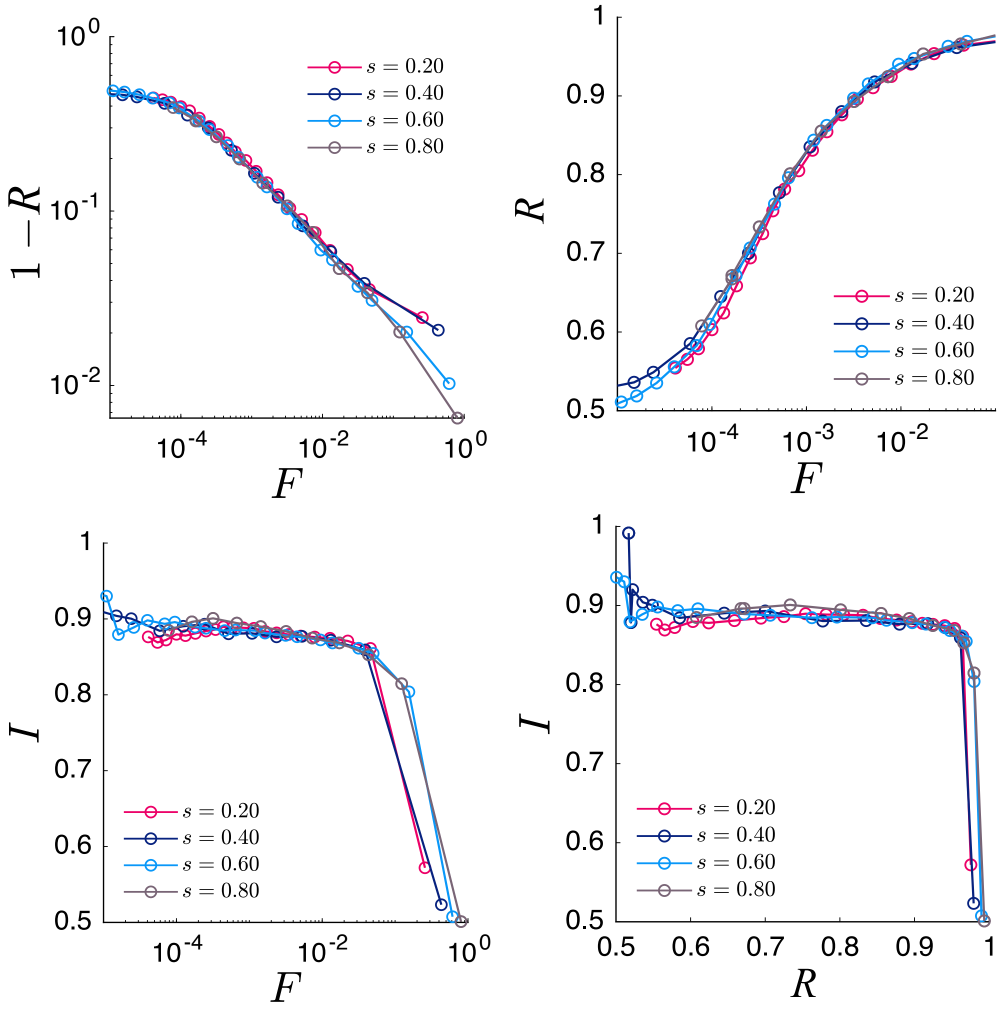

Figure 4. *Top:* On the left, logarithmic plot of $1 - R$ versus normalized committee size. On the right, R versus normalized committee size. *Bottom:* Committee Integrity as a function of normalized committee size (left) and representativity R (right). We consider a fraction of the individuals (s) which self declare as candidates (individuals in a cycle), a Erdös-Rényi network with $N_e = 10000$, $\langle k \rangle = 40$ and $\sigma^2 = \sigma_p^2 = 0.05$. Results are averaged over 100 different realizations.

References

1. Holland, P. W., Laskey, K. B., Leinhardt, S. (1983). Stochastic blockmodels: First steps. Social networks, 5(2), 109-137.
